# A protocol of randomized controlled trial for Modified Xiaoyao Powder in the treatment of chronic obstructive pulmonary disease combined mild to moderate depression

**DOI:** 10.1097/MD.0000000000023220

**Published:** 2020-11-20

**Authors:** Keling Chen, Keni Zhao, Wujun Wang, Wei Xiao, Jing Xiao, Yang Yang, Yufei Liu, Xiaohong Xie

**Affiliations:** Department of Respiratory Medicine, Hospital of Chengdu University of Traditional Chinese Medicine, Chengdu, P.R. China.

**Keywords:** chronic obstructive pulmonary disease, mild to moderate depression, Modified Xiaoyao Powder, psychotherapy

## Abstract

**Introduction:**

Depression is an important complication of chronic obstructive pulmonary disease (COPD), occurring in more than one-third of individuals with COPD, and its severity is closely related to the severity and acute exacerbation of COPD, significantly contributing to the risk of death from COPD. Comorbid depression in COPD can be a burden on COPD-related diseases by reducing quality of life and compliance with treatment. Unfortunately, symptoms of COPD combined anxiety and depression are not properly diagnosed and treated in clinical practice, especially in the early stages of mood changes in patients with COPD, as the symptoms are mild and monotonous, and are overlooked.

**Methods:**

In this prospective, randomized, placebo-controlled trial, we will assigned 280 eligible patients who had COPD combined depression to receive either Modified Xiaoyao Powder (MXP) or placebo. The primary end point is the change in the Hamilton Depression Scale (17 items) (HAMD-17) score from baseline on weeks 4, 12, and 24.

**Discussion:**

Six months of MXP for COPD combined mild to moderate depression may alleviate the symptoms of depression, reduce the frequency of hospitalizations, the number of exacerbations, and improve the compliance of treatment.

**Trial registration:**

ChiCTR2000038741.

## 1 Introductions

Chronic obstructive pulmonary disease (COPD) is defined as chronic progressive and debilitating disease with high morbidity, high disability, and high mortality,^[1]^ especially in smokers over the age of 40 years and in individuals exposed to toxic particles or gases,^[2]^ with important implications for quality of life, symptoms, comorbidities, and safety of life,^[3]^ and is a major burden of the worldwide medical economy. Chronic progressive dyspnea, cough and sputum, and irreversible airflow limitation are the main clinical manifestations and pathological features of COPD, which may lead to various adverse pulmonary and extrapulmonary effects and complications as the disease progresses, such as cardiovascular disease, skeletal muscle dysfunction, osteoporosis, anxiety and depression, diabetes mellitus, hypertension, lung cancer, etc.^[1,4]^ Statistically, >50% of COPD patients have 1 to 2 comorbidities, 15.8% have 3 to 4, and 6.8% have >5 comorbidities.^[4]^

Depression is an important complication of COPD, with a prevalence of up to 80% in the severe phase of COPD,^[5]^ and its severity is closely related to the severity and acute exacerbation of COPD,^[6,7]^ greatly associated with increased risk of mortality related COPD. At the same time, anxiety and depression in combination with respiratory illness can subjectively amplify the true perception of the illness itself, especially dyspnea or cough, leading to higher hospitalization rates and increased use of bronchodilators, inhaled and systemic corticosteroids and antibiotics, as well as the incidence of adverse events.^[8]^ To date, however, COPD guidelines do not provide definitive treatment recommendations for patients with mental disorders such as anxiety and depression. Thus, early identification, diagnosis, and treatment of COPD patients with comorbid depression is crucial.

Depression belongs to the category of “depression syndrome” in traditional Chinese medicine (TCM). There is a more systematic theoretical understanding and rich experience in diagnosis and treatment of depression syndromes, especially for mild to moderate depression.^[4]^ Liver-Qi stagnation is the most common syndrome of TCM of depression, and Xiaoyao Powder is a representative formula for the treatment of Liver-Qi stagnation. Clinical and experimental studies have shown that Xiaoyao Powder has the exact effect of improving and treating depression, with few adverse reactions,^[9]^ and that its antidepressant mechanism is related to neurotransmitters, neurotrophins, hypothalamic-pituitary-adrenal axis, amino acids, lipids, energy metabolism, and inflammatory factors.^[10]^ Therefore, the Modified Xiaoyao Powder (MXP) used in this trial will be made from Xiaoyao Powder.

Psychotherapy is considered to be the classical treatment for mild to moderate depression and is most accepted by patients. The guidelines^[11]^ also recommend TCM prescriptions with antidepressant effects for the treatment of mild to moderate depression. So, is it possible to combine the 2 treatments to achieve better clinical effects? Based on this, we designed this study, to evaluate its clinical efficacy and its effect on the prognosis of COPD.

The aim of this research project has therefore been to assess the efficacy and safety associated with MXP in treatment of mild to moderate depression.

## 2 Methods/design

### 2.1 Design

This trial will be conducted in Hospital of Chengdu University of Traditional Chinese Medicine (Chengdu, China) from December 2020 through March 2022. We will recruit patients with COPD combined mild to moderate depression and will assign them in 1:1 ratio to the treatment group or the control group. All participants will receive both personalized treatment for COPD according to GOLD 2020 guidelines and psychotherapy advocated by Chinese Expert on the diagnosis and treatment of depression with integrated traditional Chinese and western medicine. In addition, the treatment group will receive MXP and the control group will receive MXP placebo 3 times a day. The primary end point is the change in the HAMD-17 score from baseline at weeks 4, 12, and 24. The secondary end points, which will assessed at weeks 4, 12, and 24, include a reduction of >50% from baseline in the HAMD-17 score, the change from baseline in modified medical research council dyspnea scale score (mMRC score), the change from baseline in COPD assessment test (CAT score), the frequency of hospitalizations, and the number of exacerbations.

**Figure 1:**
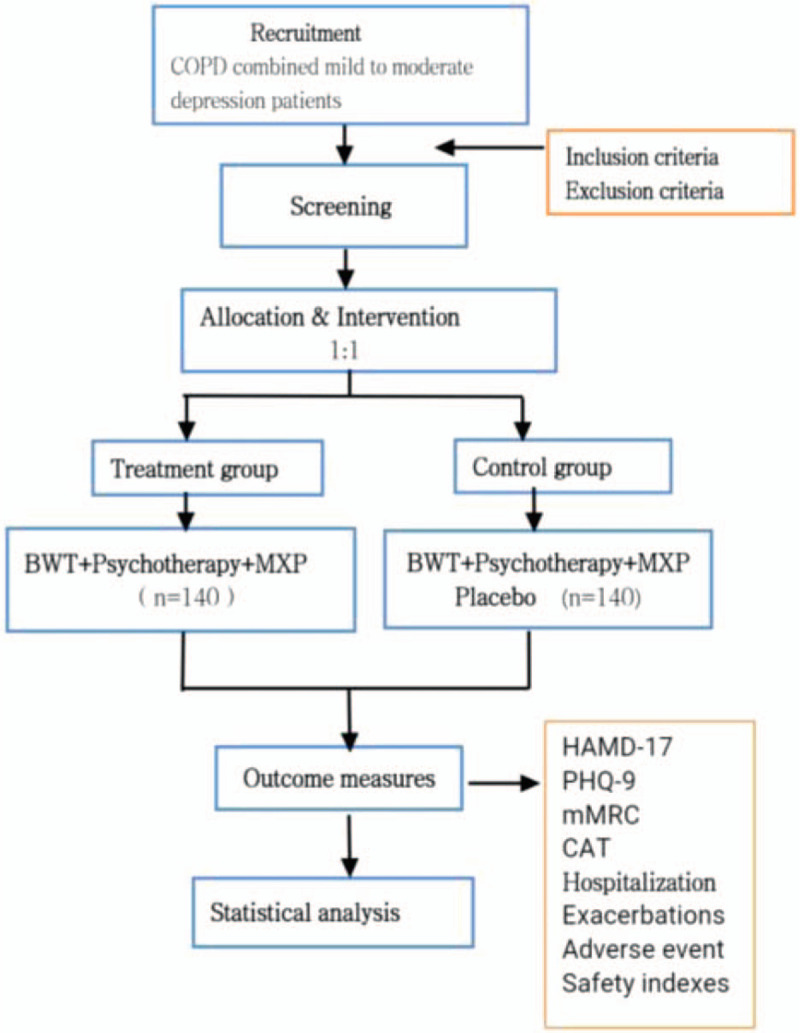
The flow chart of the study design. BWT, Basic Western medicine treatment; CAT, COPD assessment test; HAMD-17, Hamilton Depression Scale (17 items); mMRC, modified medical research council dyspnea scale scores; MXP, Modified Xiaoyao powder; PHO-9, 9-item Patient Health Questionnaire score; Safety indexes: blood, urine and feces routine, liver and renal function, electrocardiogram.

Safety will be assessed based on the times and severity of adverse events, vital signs, blood, urine and feces routine, laboratory measurements (liver and renal function, electrolyte), and electrocardiogram. Adverse events be defined as any adverse events or any deterioration of existing diseases that occurred from the first administration of MXP or MXP placebo to 7 days after the last administration. The flow chart of the study design, see Fig. 1.

### 2.2 Ethics approval

**Figure 2:**
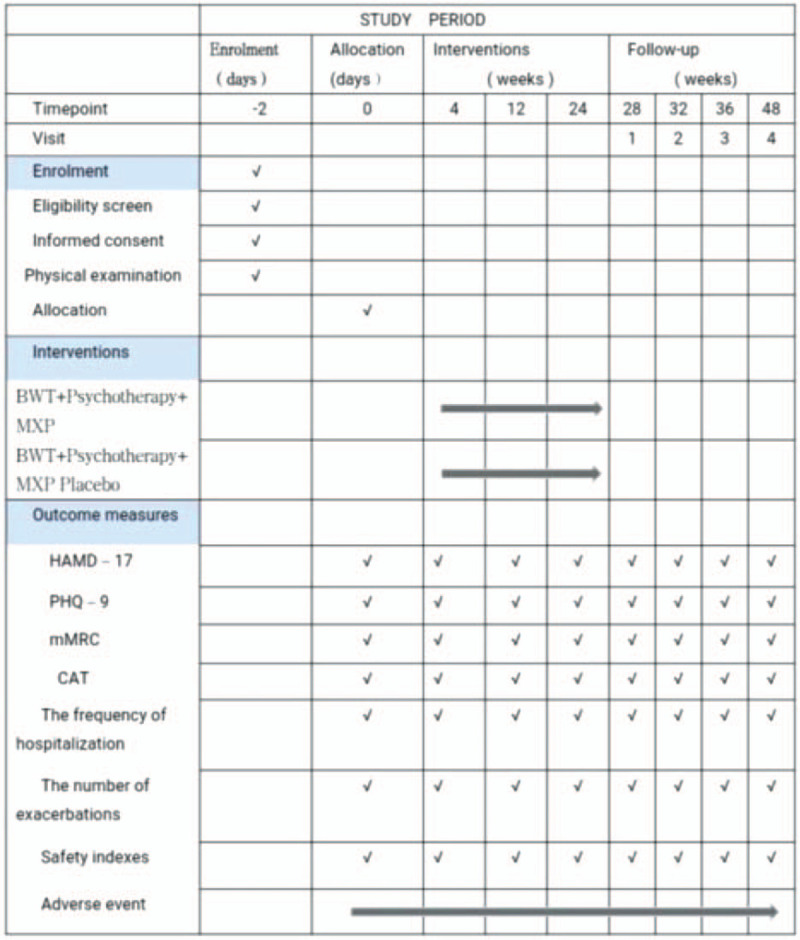
Spirit figure of enrolment, interventions, and assessments. BWT, Basic Western medicine treatment; CAT, COPD assessment test; HAMD-17, Hamilton Depression Scale (17 item); MXP, Modified Xiaoyao powder; mMRC, modified medical research council dyspnea scale scores; PHO-9, 9-item Patient Health Questionnaire score; Safety indexes: blood, urine, and feces routine, liver and renal function, electrocardiogram.

The trial registered on September 27, 2020 and was reviewed and approved by Chinese Ethics Committee of Registering Clinical Trials (Approval No. ChiECRCT20200286). This trial will be conducted in accordance with the Declaration of Helsinki and the protocol follows the recommendations of the SPIRIT 2013^[12]^ (see Additional file 1). All participants provided written informed consent. The spirit figure of enrolment, interventions, and assessments is shown below in Fig. 2.

### 2.3 Recruitment

We plan to start recruiting 280 participants, who are from the outpatient or inpatient wards of the Hospital of Chengdu University of Traditional Chinese Medicine in December 2020. The researcher takes the initiative to introduce this trial, and the participant voluntarily participate in this project. After being screened by the clinician according to the inclusion and exclusion criteria, they can be enrolled.

### 2.4 Sample size

Date from a previous study indicated that for a trial population with mild to moderate depression treated with TCM prescription with antidepressant effect for 4 weeks,^[13]^ the mean HAMD-17 score of the treatment group would be 13.33 and the standard deviation would be 4.21. The mean of the control group would be 11.57 and the standard deviation would be 5.01. We use power = 0.80, alpha = 0.05 (bilateral), a 1:1 ratio, and assuming that the drop-out rate of the trial is 20%, an estimated a total of 280 patients will be needed in this trial. PASS 15 software (NCSS Institute, LLC) will be used to calculate the sample size.

### 2.5 Randomization and concealment

A computer-controlled system implemented by the Sichuan Evidence-based Medicine Center of Traditional Chinese Medicine is randomly grouped, and a random code table corresponding to the serial number 001–280 is generated. After the participants meet the enrollment criteria and sign the informed consent, the random group number of the participants is obtained, and which is placed in a light-proof envelope and sealed. When the participant is accepted, the number is checked and unsealed therefore the participant is assigned to the group, thus, completes the treatment allocation for 280 participants. Participants and researchers cannot predict the specific grouping condition of participants.

### 2.6 Blinding

In this trial, we adopt a double-blind design, the researchers and participants are blinded without knowing the specific grouping. The dosage form, shape, taste, and color of the placebo, which consists of starch without any active ingredient, will be as close as possible to the MXP used in the treatment group. Only in emergency situations, such as a severity adverse event, or when the participant needs emergency rescue, the researcher reports to the supervisor and the principal investigator to decide whether to unblinding. Once the participant is unblinded, the case will be regarded as a dropout case and will not be included in the efficacy analysis.

### 2.7 Diagnostic criteria

Patients must meet the diagnostic criteria for COPD^[14]^ and mild to moderate depression.^[11]^

The syndrome of stagnation of Liver-Qi of depression be defined as: primary symptoms: depressed mood, oppression in chest, sighing, fullness, and distention in hypochondrium.

Secondary symptoms: abdominal distention and fullness, belching, poor appetite, breast distention before menstruation, symptoms fluctuate with mood. Tongues and pulses image: thin white coat, stringy pulse.

### 2.8 Eligibility criteria

#### 2.8.1 Inclusion criteria

- 18 to 65 years of age, include men and women.
- Patients who had received a diagnosis of COPD combined mild to moderate depression (7 ≤ HAMD-17 score ≤ 24); and meet the syndrome of stagnation of Liver-Qi of depression.
- The course of depression does not exceed 3 months.
- The patients have not taken antidepressants in the past 1 month.
- Agree to participate and sign an informed consent form.

### 2.9 Exclusion criteria

- A history of a suicide attempt, treatment-resistant depression, and familial mental illness.
- Had a recent history of other acute or chronic significant clinical manifestations medical conditions.
- A history of bipolar disorder, schizophrenia, or schizo affective disorder.
- Patients who cannot actively cooperate with the treatment.

## 3 Interventions

### 3.1 Treatment plan

All participants in this trial will be randomly divided into the treatment group or the control group. Both groups will receive individualized treatment plan for COPD referred to GOLD 2020 guidelines,^[15]^ which include:

1) A group (mMRC 0–1, CAT < 10): Setropium Bromide Powder Inhalation (Boehringer Ingelheim International GmbH 18 μg/press, registration number: H20140933) inhale, one press each time, once a day.
2) B group (mMRC ≥ 2, CAT ≥ 10): Setropium Bromide Powder Inhalation (Boehringer Ingelheim International GmbH 18 μg/press, registration number: H20140933) inhale, one press each time, once a day.
3) C group (≥2 moderate exacerbations or ≥1 leading to hospitalization): Budesonide formoterol powder inhalation (Astra Zeneca AB 160/4.5 μg/puff, registration number: H20140458) inhale, one puff each time, twice times a day.
4) D group (CAT > 20, consider if EOS ≥300): Budesonide formoterol powder inhalation (Astra Zeneca AB, 160/4.5 μg/puff, registration number: H20140458) inhale, one puff each time, twice times a day; Setropium Bromide Powder Inhalation (Boehringer Ingelheim International GmbH 18 μg/press, registration number: H20140933) inhale, one press each time, once a day.

Meanwhile, all participants will receive psychotherapy services, provided by psychosocial assistants. Those assistants must receive at least 2 weeks of face-to-face training by a qualified psychoeducation institution before the start of the trial. Only after the training can they be qualified to provide psychological services to the participants. A group mode will be adopted, with 8 to 12 patients in each group and completed by 2 psychosocial assistants. Psychotherapy services provided by psychosocial assistants to patients can be conducted face-to-face or online, 1.5 to 2 hours each time. The content adopts individualized programs based on the participant conditions, with the purpose of alleviating the symptoms of depression. During this period, the psychosocial supervisor provides direct supervision to the psychosocial assistants through telephone or interview, and inspects the work of the psychosocial assistants based on the responses of patients.

In addition, the treatment group will be provided for MXP, which produced by the Department of Pharmacy, Hospital of Chengdu University of Traditional Chinese Medicine, 1 frame each time, 3 times a day, mixed in boiled water for oral taking. The main components of MXP see Table 1.

**Table 1:** The main components of Modified Xiaoyao Powder.

Chinese name	Latin name	Amount, g
Chai hu	Radix bupleuri	15
Dang gui	Radix angelicae sinensis	12
Chi shao	Radix rubrus paeoniae lactiflorae	15
Xiang fu	Rhizoma cyperi rotundi	15
Mu dan pi	Cortex radix moutan	12
Bo he	Menthae haplocalycis	6
Bai zhu	Rhizoma atractylodes macrocephalae	15
Zhi gan cao	Radix glycyrrhizae	6
Bai he	Bulbus lilii	20
Zhi zi	fructus gardeniae jasminoidis	12
Mai ya	Fructus germinatus hordei vulgaris	12
He huan pi	Cortex albizziae julibrissinis	20

### 3.2 Outcome measures

Primary end points: The change in the HAMD-17 score from baseline (at weeks 4, 12, and 24).

Secondary end points: A reduction of >50% from baseline in the HAMD-17 score, the change from baseline in mMRC score, the change from baseline in CAT score, the frequency of hospitalizations, and the number of exacerbations (at weeks 4, 12, and 24).

Follow up: The clinical follow-up will be arranged at weeks 4, 8, 12, and 24 after the treatment. The data collected at each visit will include patient diary, HAMD-17, 9-item Patient Health Questionnaire score (PHQ-9), mMRC, CAT, hospitalization, the number of exacerbations, adverse event records, etc.

### 3.3 Data management and quality control

A dedicated data manager will be assigned for data entry and management. The data administrator will use EpiData software, version 3.1 (The EpiData Association, Odense, Denmark) for data entry and management. To ensure data accuracy, double entry and proofreading should be done by 2 data administrators independently. The data administrator can send queries to the investigator through the clinical supervisor in the case report form (CRF), and the investigator should answer and return the queries as soon as possible. After reviewing and confirming that the database created is correct, the data are locked by the project host unit, principal investigator, and statistical analysts. No further changes will be made to the data or files after the locking. Any problems found after the data lock is confirmed and corrected in the statistical analysis program.

### 3.4 Statistical analysis

Use SAS software version 9.3 (SAS Institute, Cary, NC) for statistical analysis. Continuous variables are expressed as mean and standard deviation, and categorical variables as numerical values and percentages. The chi-square test and *t* test will be used to compare baseline characteristics between the study groups. Logistic regression analysis will be used to determine factors related to follow-up failure. The repeated measures mixed effects model will be used to analyze the least squares mean changes of HAMD-17, PHQ-9, CAT, mMRC scores from baseline. The generalized estimation equation model will be used to analyze the categorical variable HAMD-17 score for baseline reduction >50%, the number of exacerbations, and the frequency of hospitalizations. Efficacy analysis will be performed in accordance with the principle of intention-to-treat (including all patients included in the randomized group, excluding excluded cases). For missing data, the method of sequence average will be used to replace missing data.

All hypothesis adopts 2-sided testing, and *P* < .05 is statistically significant.

## 4 Discussion

As a chronic pulmonary disease, COPD has a long duration, recurrent episodes, and is often associated with mental states such as anxiety and depression. Patients with COPD and depression often present clinically with depressed mood, lack of interest, fatigue, anorexia, sleep disturbances, and other cognitive, behavioral, and social abnormalities. Increased the number of acute exacerbations and hospitalizations reduce the quality of life of COPD patients. Prevalence studies have shown that patients with COPD are 4 times more likely to suffer from depression than those without COPD. The prevalence is especially high in the severe stage of COPD, making it an important challenge in the treatment of COPD.

TCM has a long history and rich experience in treating depression, and is characterized by bidirectional regulation, multitarget, multisystem, and multilevel in antidepressants. Therefore, the combination of TCM with psychotherapy for improving depressive symptoms in patients with mild to moderate depression and achieving the optimal combination with few adverse effects is the most important feature of this trial design, as previous studies have confirmed the clear efficacy of TCM for mild to moderate depression. Although the combined pharmacological and psychological treatment approach is not the first innovation in this trial, the TCM prescriptions and outcome indicators selected in this trial can provide more evidence for the clinical treatment of depression from various aspects and perspectives.

Secondly, complications are not conducive to the treatment and prognosis of COPD patients. Therefore, at the beginning of the design of this trial, this factor was taken into consideration, and the specific efficacy of early intervention on the prognosis of patients with COPD will be measured by assessing the number of acute exacerbations, hospitalization rate, and clinical symptoms of COPD.

Furthermore, treatment based on syndrome differentiation is the characteristic of TCM. The same disease may manifest itself in multiple syndromes, and the selection of the TCM prescription is also different. Thus, the shortcoming of this trial is that in actual clinical practice, the TCM diagnosis of patients with COPD combined depression is not limited to the single syndrome of Liver-Qi stagnation, so the TCM prescriptions selected in this trial are not applicable to all patients with depression. Besides, due to the single-center design, the sample size of this trial was limited.

In a word, in our study, TCM combined with psychotherapy, as compared with single treatment, will be effective in reducing depression and anxiety symptoms and improve COPD patients prognosis.

## 5 Others

### 5.1 Trial status

This trial will began from December, 2020, and approximate date of completion is March, 2022. A total 280 participants will be enrolled.

### 5.2 Funding

The study supported by Sichuan Science and technology program (2020JDRC0114 2020YFH0164). The funder has provided only financial support for the study.

### 5.3 Consent for publication

Written informed consent will be taken from all participants at the start of recruitment in this study.

## Acknowledgments

Thanks to Dr. Chuantao Zhang for his assistance and valuable advice.

## Author contributions

**Conceptualization:** Wei Xiao, Yufei Liu.

**Formal analysis:** Yang Yang.

**Investigation:** Wei Xiao.

**Supervision:** Xiaohong Xie.

**Writing – original draft:** Keling Chen, Keni Zhao.

**Writing – review & editing:** Keling Chen, Keni Zhao, Wujun Wang, Jing Xiao.

## Supplementary Material

Supplemental Digital Content
